# MCMC implementation of the optimal Bayesian classifier for non-Gaussian models: model-based RNA-Seq classification

**DOI:** 10.1186/s12859-014-0401-3

**Published:** 2014-12-10

**Authors:** Jason M Knight, Ivan Ivanov, Edward R Dougherty

**Affiliations:** Department of Electrical Engineering in Texas A&M University, 3128 TAMU, College Station, 77843 TX USA; Department of Veterinary Physiology and Pharmacology in Texas A&M University, 3128 TAMU, College Station, 77843 TX USA; Center for Bioinformatics and Genomics Systems Engineering, Texas A&M University, College Station, 77843 TX USA

**Keywords:** Classification, RNA-Seq, Model-based, Bayesian

## Abstract

**Background:**

Sequencing datasets consist of a finite number of reads which map to specific regions of a reference genome. Most effort in modeling these datasets focuses on the detection of univariate differentially expressed genes. However, for classification, we must consider multiple genes and their interactions.

**Results:**

Thus, we introduce a hierarchical multivariate Poisson model (MP) and the associated optimal Bayesian classifier (OBC) for classifying samples using sequencing data. Lacking closed-form solutions, we employ a Monte Carlo Markov Chain (MCMC) approach to perform classification. We demonstrate superior or equivalent classification performance compared to typical classifiers for two synthetic datasets and over a range of classification problem difficulties. We also introduce the Bayesian minimum mean squared error (MMSE) conditional error estimator and demonstrate its computation over the feature space. In addition, we demonstrate superior or leading class performance over an RNA-Seq dataset containing two lung cancer tumor types from The Cancer Genome Atlas (TCGA).

**Conclusions:**

Through model-based, optimal Bayesian classification, we demonstrate superior classification performance for both synthetic and real RNA-Seq datasets. A tutorial video and Python source code is available under an open source license at http://bit.ly/1gimnss.

**Electronic supplementary material:**

The online version of this article (doi:10.1186/s12859-014-0401-3) contains supplementary material, which is available to authorized users.

## Background

The possibility of genomic phenotype classification arose with the inception of gene-expression microarrays. From the outset, two fundamental problems have frustrated the endeavor: (1) the inaccuracy of microarray measurements, and (2) small samples. Our particular application of interest is classification using RNA-Seq data. Modern RNA-Seq technologies sequence small RNA fragments (mRNA) to measure gene expression, where the number of reads mapped to a gene on the reference genome defines the count data. Given that RNA-Seq data has advantages over microarray data, in particular, more accurate measurement, we still confront the second fundamental problem, which is statistical, not technological: small samples cause re-sampling-based classifier error estimators to be very inaccurate due to excessive variance and lack of regression with the true error [1-4]. Since the error rate of a classifier quantifies its predictive accuracy, it is the salient epistemological attribute of any classifier. The inability to satisfactorily estimate the error with model-free methods with small samples implies that genomic classifier error estimation is virtually impossible without the use of prior information, so that the whole small-sample classification problem becomes unapproachable in a model-free framework [5].

The situation has been addressed by utilizing prior knowledge via a Bayesian approach that considers a prior distribution on an uncertainty class of feature-label distributions [6,7]. For expression-based classification, prior distributions have been constructed using expression data not employed in classifier design [8] and known regulatory pathways [9]. Given that a prior model must be assumed to achieve satisfactory error estimation, an obvious course of action is to derive an optimal classifier based on the prior knowledge and the sample data, the result being an optimal Bayesian classifier (OBC) that is guaranteed to have the best average performance of any classifier relative to the posterior distribution derived from the prior distribution and data [10,11]. While Bayesian classification does not depend on particular distributional forms, closed-form solutions have been derived for the multinomial model and Gaussian models using linear classifiers for the minimum mean squared error (MMSE) error estimate [6,7], the MSE of the error estimate [12,13], and an optimal Bayesian classifier (OBC) relative to the prior distribution [10,11], the latter being expressed in terms of *effective class conditional distributions*, which are expectations relative to the posterior distribution of the class-conditional distributions. The closed-form solutions depend on particular models (multinomial and Gaussian) and the existence of conjugate priors, which can be too constraining for practical applications such as RNA-Seq classification.

Much of the statistical literature concerning classification of RNA-Seq data attempts to address differential expression testing, that is, univariate statistical testing on an individual gene basis. These attempts typically model RNA-Seq data via negative binomial [14,15] and Poisson distributions [16]. In addition, network inference has been attempted using a hierarchical Poisson log-normal model [17], and clustering of RNA-Seq data points has utilized various approaches [18,19]. However, in clinical settings one is often interested in sample classification: the problem of classifying the RNA-Seq data from unlabeled patients using a set of labeled training data. One of the few RNA-Seq-specific attempts towards this goal uses a Poisson modeling assumption with independent features [20]. The Poisson model is completely parameterized by its mean and thus is known to exhibit problems in fitting RNA-Seq data due to the overdispersion typically observed in such datasets.

In this paper, we focus on modeling the pipeline that starts with extracting the gene concentrations from the biological samples and their subsequent processing by the sequencing instrument [21]. This is accomplished using a hierarchical, multivariate Poisson model (MP). Specifically, gene concentration levels are modeled by a log-normal distribution and the sequencing instrument sampling of those is modeled via a Poisson process. This allows us to accurately model the RNA-Seq data overdispersion as demonstrated by marginal variance calculations and posterior predictive model diagnostics in Section ‘Overdispersion’. In addition, this hierarchical model allows for inferring any covariance structure observed between the features.

Whereas Dalton and Dougherty have presented a computational method for nonlinear classifiers in the Gaussian model [8], this still depends upon conjugate priors. In this work, we remove the constraints imposed by the requirement of a closed-form solution by developing the optimal Bayesian classifier using a Markov-chain-Monte-Carlo (MCMC) methodology. This provides a computational framework for calculating the OBC for any parameterized class conditional-density and any prior distribution. Most notably, this allows us to use distributions designed to closely model particular datasets and a prior distribution of any form to improve classification performance in small-sample settings, in particular, for RNA-Seq-based classification.

## Methods

### Notation

Throughout, we use capital letters to indicate random variables, lower case letters to indicate individual realizations of random variables or indices, bold latin characters for observed vectors, and Greek letters for latent features and parameters. We write *p*(**X**) as the probability measure over the random variable **X**. *p*(**X**) may be a probability mass function, probability density function, or arbitrary probability measure. *p*(**x**|*y*) denotes the conditional probability *p*(**X**=**x**|*Y*=*y*). Similarly, following Bayesian convention, we write parameterized distributions by conditioning on the parameter, for instance, *p*(**X**|*Y*,*θ*), and posterior expectations by conditioning on the sample, such as *E*[**X**|*Y*,*S*_*n*_], where *S*_*n*_ and all other values are defined in Section ‘Review of optimal Bayesian classification’. If it is unclear which density an expectation is taken with respect to, then we denote it in subscript notation, such as $E_{\theta |S_{n}}\left [\cdot \right ],$ where the expectation is taken with respect to the density *p*(*θ*|*S*_*n*_).

### Review of optimal Bayesian classification

Binary classification considers a set of *n* labeled training data points, $S_{n}=\left \{(\mathbf {x},y)\right \}_{1}^{n}$, where *y*∈{0,1} is the class label and $\mathbf {x}\in \mathcal {X}$ is the feature vector over a feature space . An example of binary classification in a clinical setting might include class 0 and 1 being two types of cancers, or normal and cancerous tissues. Available features would then be the gene or genes that will eventually be used in the designed classifier to assign this label. The feature space  would be the set of possible gene expression measurements for all genes in the feature vector. The labeled training data *S*_*n*_ would be the set of gene expression measurements from samples which had undergone further testing (possibly observation with the passage of time, cell culturing, or more invasive followup procedures) to identify the type or malignancy of the tissue. Using *S*_*n*_, we design a classifier *ψ* that hopefully performs well on the unknown joint feature-label distribution *p*(**X**,*Y*). In the same clinical example, the classifier *ψ* could then identify the type of cancer using gene expression measurements alone.

By parameterizing this unknown joint distribution in a model-based Bayesian framework one can derive an optimal Bayesian classifier (OBC) that minimizes the expected error over the space of all classifiers under assumed forms of the class-conditional densities. Specifically, under Gaussian and multinomial class-conditional densities and their corresponding conjugate prior distributions, closed-form solutions for the OBC [10,11] and the first two moments of the error estimate conditioned on the sample [12,13] have been obtained.

The parameterization of the feature-label distribution consists of the marginal class probability *c* and the class-conditional densities *p*(**x**|*y*,*θ*_*y*_), where a particular value *θ*_*y*_∈*Θ*_*y*_ specifies a single class-conditional density contained in the class of densities defined over the space *Θ*_*y*_, which will be a Cartesian product as described in Section ‘The multivariate poisson model’. Therefore, for a two-class problem, we specify a parameterized joint feature-label distribution as *θ*=(*c*,*θ*_0_,*θ*_1_)∈*Θ*=[0,1]×*Θ*_0_×*Θ*_1_. In the Bayesian classification framework, these values are then treated as random variables, so that we may consider quantities such as the expectation of *c*, or another random variable conditioned on the value of the parameter vector *θ*.

**Figure 1:**
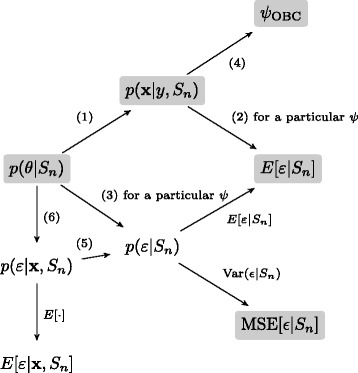
**Bayesian classification derivation tree.** A tree summarizing the relationships between several important quantities in the general theoretical framework of Bayesian classification. A directed edge between a parent and its child indicates that the child can be derived from the parent by the equations indicated in the edge label. The root of the tree *p*(*θ*|*S*_*n*_) is the posterior distribution of the feature label parameters and by taking expectations with respect to this distribution, we can derive the effective class conditional densities *p*(**x**|*y*,*S*_*n*_) and the distribution of the classifier error *p*(*ε*|*S*_*n*_). Then these quantities give rise to the OBC, and MMSE and MSE estimates for the error as described in the text. Quantities highlighted in grey are given in closed form for Gaussian and multinomial distributions in [12].

Figure 1 describes the inter-relationships between the quantities of interest in the general theoretic framework of Bayesian classification. The tree shows a subset of the derivations possible from the posterior feature-label parameter distribution to the OBC classifier and error estimates. Specifically, directed edges indicate that the child can be derived from the parent by performing the operation indicated by the edge label. Closed-form solutions of the quantities highlighted in grey have been calculated for the Gaussian and multinomial feature-label distributions [6,7]. As in those derivations, the tree assumes independence between the marginal class probability *c* and the class-conditional parameters *θ*_*y*_. In addition, the posterior of *c* is assumed known throughout the tree. Figure 1 demonstrates a primary benefit of the Bayesian approach to classification. Once we obtain the posterior distribution of the class-conditional parameters, it is straightforward to calculate many relevant quantities through appropriately crafted conditional expectations. In this paper we demonstrate how to approximate any quantity in the tree for arbitrary class conditional densities and arbitrary prior distributions.

We now examine the tree in more detail. Starting at the far left of the tree, *p*(*θ*|*S*_*n*_) is the posterior distribution of the parameterized feature-label distribution – posterior to the labeled samples in *S*_*n*_. Typically, error estimates and the optimal classifier are our primary interest, so that this posterior distribution is traditionally used as a means to compute other quantities and is not of interest by itself.

The *effective class-conditional density* is the marginal predictive posterior of the feature vector **X** conditioned *S*_*n*_ and the class variable *Y*,

(1) $$ p\left(\mathbf{x}|y,S_{n}\right)=\int_{\Theta_{y}}p\left(\mathbf{x}|y,\theta_{y}\right)p\left(\theta_{y}|S_{n}\right)d\theta_{y}.  $$

It gives the distribution of the feature vector using a weighted average over all the parameterized class-conditional densities in *Θ*_*y*_ given a class *y*. The weights in this expectation are the posterior, *p*(*θ*_*y*_|*S*_*n*_), evaluated at each *θ*_*y*_.

The true error of classifier *ψ* is *ε*=*p*(*ψ*(**X**)≠*Y*). Given the sample data *S*_*n*_, *ε* is a random unknown quantity in the Bayesian framework. The MMSE estimate given in [12] can be written as

(2) $$\begin{array}{@{}rcl@{}} E\left[\varepsilon |S_{n}\right]& =&p\left(\psi (\mathbf{X})\neq Y|S_{n}\right) \\ & =&E_{\theta |S_{n}}\left[p\left(\psi(\mathbf{X})\neq Y|\theta,S_{n}\right)\right] \\ & =&\hat{c}\varepsilon_{0}\left(\theta_{0},\psi\right)+(1-\hat{c})\varepsilon_{1}\left(\theta_{1},\psi\right) \\ & =&\int_{\mathcal{X}}\left(\hat{c}p\left(\mathbf{x}|0,S_{n}\right)\mathbf{I}_{\mathbf{x}\in R_{1}}\right. \\ &&\left. +\;(1-\hat{c})p\left(\mathbf{x}|1,S_{n}\right)\mathbf{I}_{\mathbf{x}\in R_{0}}\right)d \mathbf{x},  \end{array} $$

where **I**_*A*_ is the indicator function for event *A*, $\hat {c} =E\left [c|S_{n}\right ]$ is the posterior expectation of *c*, *R*_*y*_ is the region of the feature space the classifier predicts to be class *y*,  is the feature space, and *ε*_*y*_(*θ*_*y*_,*ψ*) is the error of classifier *ψ* contributed by class *y* on the fixed distribution *θ*_*y*_.

We can also obtain the full posterior distribution of the error,

(3) $$\begin{array}{@{}rcl@{}} p\left(\varepsilon |S_{n}\right)& =&\int_{\Theta}p\left(\varepsilon |\theta\right)p\left(\theta |S_{n}\right)d\theta \\ & =&E_{\theta |S_{n}}\left[p\left(\varepsilon |\theta\right)\right],  \end{array} $$

where *p*(*ε*|*θ*) is the true error for a fixed feature-label distribution and fixed classifier. We denote this deterministic function by *ε*(*θ*,*ψ*). As shown in Figure 1, the MMSE estimate and the sample conditioned MSE for this error can also be calculated using the first two moments of the error distribution.

With the MMSE estimator defined, the optimal Bayesian classifier (OBC) is the classifier minimizing the expected error by pointwise minimization of the integral (2) [11]:

(4) $$ \psi_{\text{OBC}}(\mathbf{x}) = \left\{ \begin{array}{lll} 0 && \text{if }\hat{c}p(\mathbf{x}|0,S_{n})\geq (1-\hat{c})p(\mathbf{x} |1,S_{n}), \\ 1 && \text{otherwise.} \end{array}\right..   $$

### Conditional error estimator

If the true feature-label distribution were known, then we could compute the true error of a classifier exactly as an expectation over the conditional error [22]:

$$\varepsilon =p(\psi (\mathbf{X})\neq Y)=\int_{\mathcal{X}}p(\psi (\mathbf{x})\neq Y|\mathbf{x})p(\mathbf{x})d\mathbf{x}. $$

Treating *ε* as a random variable, one can similarly derive its posterior distribution by conditioning on the feature vector:

(5) $$\begin{array}{@{}rcl@{}}{} p\left(\varepsilon |S_{n}\right)& =&\int_{\mathcal{X}}p\left(\varepsilon,\mathbf{x}|S_{n}\right)d \mathbf{x} \\ & =&\int_{\mathcal{X}}\int_{\Theta }p\left(\varepsilon,\theta,\mathbf{x} |S_{n}\right)d\theta d\mathbf{x}  \\ & =&\int_{\Theta }p\left(\theta |S_{n}\right)\int_{\mathcal{X}}p\left(\varepsilon |\mathbf{x},\theta \right)p\left(\mathbf{x}|S_{n}\right)d\mathbf{x}d\theta, \end{array} $$

which is different than the derivation of the same quantity in (3).

This introduces the idea of the *conditional error estimator,* which we define as the MMSE estimate of the classification error conditioned on the feature vector **x**,

(6) $$\begin{array}{@{}rcl@{}}{} \hat{\varepsilon}(\psi,\mathbf{x})& =&E_{\theta |S_{n}}\left[\varepsilon |\mathbf{x},S_{n}\right] \\ & =& p \left(\psi (\mathbf{x})\neq Y|\mathbf{x},S_{n}\right) \\ & =& \frac{p\left(\mathbf{x}|Y\neq \psi (\mathbf{x}),S_{n}\right)p\left(Y\neq \psi (\mathbf{x})|S_{n}\right)}{p\left(\mathbf{x}|S_{n}\right)}\\ & =& Z^{-1} p\left(\mathbf{x}|Y\neq \psi (\mathbf{x}),S_{n}\right)p\left(Y\neq \psi (\mathbf{x})|S_{n}\right), \end{array} $$

as expanded through application of Bayes’ theorem, where *Z* is a normalizing constant given by

$$Z=p\left(\mathbf{x}|S_{n}\right)=\sum_{y\in \left\{0,1\right\}}p\left(\mathbf{x}|y,S_{n}\right)p\left(y|S_{n}\right). $$

In addition to being useful in the above alternative derivation of the classifier’s error posterior, the conditional error estimate has other practical applications. When classifying an unlabeled data point, we would like to estimate the error of the classifier output for that particular data point, as opposed to the overall error estimate for the classifier.

For the OBC, from (4) the conditional error estimator can be written as

(7) $$ \hat{\varepsilon}\left(\psi_{\text{OBC}},\mathbf{x}\right)=Z^{-1}\min_{y\in\left\{0,1\right\}}\left\{ p\left(\mathbf{x}|y,S_{n}\right)p\left(y|S_{n}\right)\right\}.  $$

In sum, using the effective class-conditional densities and the posterior marginal probabilities one can calculate conditional error estimates for points in the feature space in addition to the earlier quantities described.

### The multivariate poisson model

With the widespread use of next-generation sequencing techniques, classification approaches must be developed to account for the discrete nature of the mapped sequence data and to accommodate the various types of prior information available regarding these experiments.

Gene concentration levels can be modeled using a log-normal distribution [23,24]. As discussed in the introduction, we assume that the sequencing instrument samples this mRNA concentration through a Poisson process and obtains *X*_*i*,*j*_ reads for sample point *i* and gene *j*. We model this as

(8) $$ p\left(X_{i,j}|\lambda_{i,j}\right)\sim \text{Poisson}\left(d_{i}\exp\left(\lambda_{i,j}\right)\right),   $$

where *λ*_*i*,*j*_ is the location parameter of the log-normal distribution for sample *i* and gene *j*, and *d*_*i*_ is a variable accounting for the sequencing depth as determined by the sequencing process [21]. For each *i*, we model the location parameter vector *λ*_*i*_ with a multivariate Gaussian distribution, *λ*_*i*_∼Normal(*μ*,*Σ*). We then consider the mean *μ* and covariance *Σ* of the gene concentrations as independent quantities for each class *y*.

**Figure 2:**
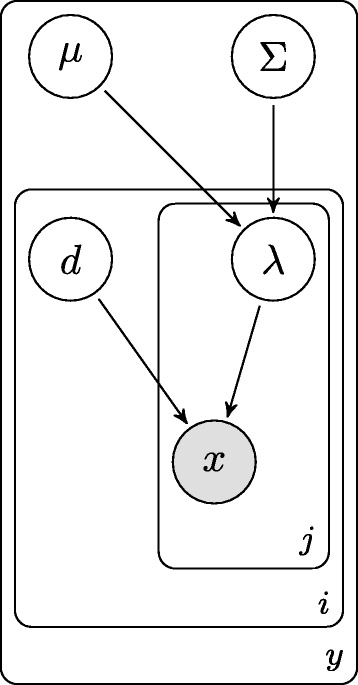
**Multivariate poisson model plate diagram.** A plate diagram for the multivariate Poisson model. The outermost plate represents the classes that we are interested in classifying against, where *i* is the index of the sample in class *y*, and *j* are the genes being modeled.

The entire MP model is represented in Figure 2 as a plate diagram. The distribution of a single class *y* is parameterized by *θ*_*y*_=(*μ*,*Σ*,**d**,**λ**), where **d**=(*d*_1_,…,*d*_*n*_) and **λ**=(*λ*_*i*,*j*_),*i*=1,2,…*n*,*j*=1,2,…,*D*, for *n* sample points and *D* total genes. Therefore, $\theta _{y}\in \Theta _{y}=\mathbb {R}^{D}\times \mathbb {R}^{D\times D}\times \mathbb {R}^{n}\times \mathbb {R}^{D\times n}$. The feature-label distribution parameterization for the two-class problem is then given by *θ*=(*c*,*θ*_0_,*θ*_1_), where *c*=*p*(*Y*=0), the prior probability for class 0.

To ensure a proper posterior with unit integral, we place weakly informative priors over the latent variables in the MP model. In choosing these values, we have aimed to avoid the complications that can occur with overly diffuse priors, such as Lindley’s paradox [25,26]. We choose:

$$\begin{aligned} \mu_{y}& \sim \text{Normal}\left(\eta_{y},\nu^{2}I_{D}\right) \\ \Sigma_{y}& \sim \text{Inverse-Wishart}\left(\kappa_{y},S_{y}\right) \\ c& \sim \text{Beta}(1,1), \end{aligned} $$

where each element of *μ*_*y*_ is distributed according to a univariate Gaussian. Unless otherwise stated, *η* is the *D* dimensional zero vector, *ν*^2^=25, *κ*=10, and *S*=(*κ*−1−*D*)*I*_*D*_. For computational and identifiability reasons, **d** is fixed to be a vector of normalization constants in order to match the different sequencing depths across all the samples. In practice, **d** can be approximated by an upper quartile normalization, which has been shown to be effective [27].

In any Bayesian approach the choice of prior affects the results, especially when only a few data points are given. In the case of MMSE classifier error estimation in the Bayesian framework, robustness to incorrect modeling assumptions has been extensively studied in [7] and in those studies performance held up well for various kinds of incorrect modeling assumptions. Robustness of optimal Bayesian classifiers to false modeling assumptions was extensively studied in [11]. Again, good robustness was exhibited. Of course, one can get bad small-sample results by intentionally selecting an inaccurate prior. In general, if one is confident in his knowledge, then a tight prior is called for because tighter priors require less data for good performance; on the other hand, when one is not confident, then prudence calls for a less informative prior. As proven in [11], OBC classification is consistent under very general conditions; however, a prior whose mass is concentrated far away from the true parameters will perform worse than one that is non-informative. These issues have been extensively discussed in the Bayesian literature [9,28-30]. In the end, performance is the measure of worth and our results with synthetic and real data indicate solid performance for the modeling approach used herein.

### Overdispersion

The MP model uses the Poisson distribution in a hierarchical scheme. It is important to note that, while the read counts are modeled as *conditionally* Poisson in equation 8, the observed read counts are not *marginally* Poisson distributed. To demonstrate this, consider a one-dimensional simplification of the MP model in which *X* is the number of reads observed, *λ* is the log of the RNA concentration, and

$$\begin{aligned} \lambda &\sim \text{Normal}\left(\mu, \sigma^{2}\right) \\ X &\sim \text{Poisson}(\exp(\lambda)). \end{aligned} $$

Then for the marginal variance of *X*,

$$\begin{aligned} \text{Var}(X) &= \text{E}\left[\text{Var}(X|\lambda)\right] + \text{Var}(\text{E}\left[X|\lambda\right])\\ &= e^{(\mu + \sigma^{2}/2)} +(e^{\sigma^{2}} - 1)e^{(2\mu + \sigma^{2})} \\ &\geq e^{\mu} = \text{Var}\left(\text{Poisson}\left(e^{\mu}\right)\right) \end{aligned} $$

where *μ* and *σ*^2^ are the mean and variance of the log of the concentration. Therefore, when *σ*^2^>0, the marginal variance of *X* is always greater than that of a Poisson random variable with the same effective rate.

In addition, by carrying out a posterior predictive model check [31], p. 143, by computing marginal posterior p-values against real RNA-Seq data, we can quantitatively assess the ability of the MP model to fit the dispersion of the TCGA data. For a test statistic *T*, we compute the p-value by comparing the test statistic on the true data *T*(*S*_*n*_) and the value of the statistic averaged across the posterior predictive distribution *T*(*x*^*rep*^), where *x*^*rep*^∼*p*(*x*|*S*_*n*_):

$$\begin{aligned} p_{T}& =\text{Pr}(T(x^{rep})\geq T(S_{n})|S_{n}) \\ & = \text{Pr}(T(x^{rep})>T(S_{n})|S_{n}) \\ & \quad+\; (0.5)\text{Pr}(T(x^{rep})=T(S_{n})|S_{n}) \\ & \approx \frac{1}{M}\sum_{i=0}^{M}\mathbf{I}\{T(x^{rep(s)})>T(S_{n})\} \\ & \quad+\; 0.5\mathbf{I}\{T(x^{rep(s)})=T(S_{n})\}, \end{aligned} $$

where *x*^*rep*(*s*)^ are Monte Carlo samples taken from the posterior predictive distribution *p*(*x*|*S*_*n*_) using the *M* Monte Carlo samples from the posterior distribution of *θ* as described in Section ‘Computation’. The term (0.5)Pr(*T*(*x*^*rep*^)=*T*(*S*_*n*_)|*S*_*n*_) is necessary due to the discrete nature of RNA-Seq data. P-values away from 0 and 1 indicate that the model posterior produces test statistics both above and below that measured on the real data.

We also consider where the real test statistic falls in relation to credible intervals of the test statistic to consider the magnitude of any differences. We apply the inter-quartile distance test statistic to provide a measure of the MP model’s ability to fit the dispersion of RNA-Seq data. We also consider several other test quantities in the Additional file 1: Table S1-S5.

### Prior calibration using discarded features

Since designed classifiers typically use very few of the totality of observed genes, only a small fraction of the data is used for classifier design. Similarly to [8], we can use the discarded features to calibrate the inverse-Wishart prior for our MP OBC. Our goal is to obtain hyperparameters *S*,**m**,*κ*, and *ν*^2^ for each class from our training data *S*_*n*_. In general, we do not expect the discarded features to give us information about any particular genes and the specific covariances between genes, so we make the simplifying assumptions that we learn information from the discarded genes in an aggregate sense. Thus, we consider the following structure on the hyperparameters: **m**=*m*[1,1,…,1]^*T*^ and



where $m\in \mathbb {R}$, *σ*^2^>0, and −1≤*ρ*≤1. For each class, we need to determine values for five scalar quantities: *m*,*ν*^2^,*σ*^2^,*ρ*, and *κ*.

Due to the hierarchical design of the MP model, we cannot apply the method of moments in a direct fashion, as did [8]. Instead, we utilize a sampling based approach to the method of moments. This MCMC sampling approach has been examined in [32] as an extension to the generalized method of moments [33]. The sampling approach uses the discarded features in an additional MCMC run evaluated prior to the primary classification MCMC procedure as discussed in Section ‘Computation’ – and then proceeds to the method of moments. In this calibration MCMC, we initialize all prior distributions with flat priors and use the discarded features to obtain samples from the posterior distribution of *μ* and *Σ*. Typically, the number of discarded features *F* is much larger than the dimensionality *D* of the classification problem. Therefore, due to computation time, we uniformly sample *F*_*s*_ pairs of features from *F* and average the resulting runs rather than using all or large groups of discarded features in a single MCMC run. We use the following procedure (for the complete algorithm, see Additional file 1):

1. For each randomly chosen discarded feature pair (*s* in total):
  - Obtain MCMC samples using the feature pair as data and flat priors.
  - Record posterior averages of *μ* and *Σ*.
2. Average over these posterior averages as given by Equations (15)- (19).
3. Using the resulting five hyperparameter estimates, run the final MCMC for classification.

Following [8], we use the moments of the posterior samples to determine the hyperparameters through the following relations: The mean of an inverse-Wishart distribution is

(9) $$ \text{E}\left[\Sigma\right]=\frac{S}{\kappa -D-1},   $$

which together with our simplified covariance structure implies

(10) $$\begin{array}{@{}rcl@{}} \sigma^{2}& =&\left(\kappa -D-1\right)\text{E}\left[\Sigma_{11}\right], \end{array} $$

(11) $$\begin{array}{@{}rcl@{}} \rho & =&\frac{\text{E}\left[\Sigma_{12}\right]}{\text{E}\left[\Sigma_{11}\right]}. \end{array} $$

The variance of the first diagonal of an inverse-Wishart matrix can be used to solve for *κ* via

(12) $$ \kappa =\frac{2\left(\text{E}\left[\Sigma_{11}\right]\right)^{2}}{\text{Var}\left(\Sigma_{11}\right)}+D+3.  $$

As we have samples of *μ* directly from our posterior, we obtain

(13) $$\begin{array}{@{}rcl@{}} m & =&\text{E}\left[\mu_{1}\right], \end{array} $$

(14) $$\begin{array}{@{}rcl@{}} \nu & =&\text{Var}\left[\mu_{1}\right]. \end{array} $$

In order to use Equations (9)-(14), we obtain estimates of the moments from MCMC performed over the *F*_*s*_ discarded feature pairs. For the *i*-th feature pair we obtain the posterior means $\widehat {\mu }_{1}^{(i)},\widehat {\Sigma }_{11}^{(i)},$ and $\widehat {\Sigma }_{12}^{(i)}$ and then average:

(15) $$\begin{array}{@{}rcl@{}} \widehat{\text{E}}\left[\mu_{1}\right]& =&\frac{1}{F_{s}}\sum_{i=1}^{F_{s}}\hat{\mu} _{1}^{(i)}  \end{array} $$

(16) $$\begin{array}{@{}rcl@{}} \widehat{\text{Var}}\left[\mu_{1}\right]& =&\frac{1}{F_{s}-1}\sum_{i=1}^{F_{s}}\left(\widehat{\text{E}}\left[\mu_{1}\right]-\hat{\mu}_{1}^{(i)}\right)^{2} \end{array} $$

(17) $$\begin{array}{@{}rcl@{}} \widehat{\text{E}}\left[\Sigma_{11}\right]& =&\frac{1}{F_{s}}\sum_{i=1}^{F_{s}}\frac{ \hat{\Sigma}_{11}^{(i)}+\hat{\Sigma}_{22}^{(i)}}{2} \end{array} $$

(18) $$\begin{array}{@{}rcl@{}} \widehat{\text{E}}\left[\Sigma_{12}\right]& =&\frac{1}{F_{s}}\sum_{i=1}^{F_{s}}\hat{ \Sigma}_{12}^{(i)} \end{array} $$

(19) $$\begin{array}{@{}rcl@{}} \widehat{\text{Var}}\left[\Sigma_{11}\right]& =&\frac{1}{F_{s}-1}\sum_{i=1}^{F_{s}}\left(\widehat{\text{E}}\left[\Sigma_{11}\right]-\hat{\Sigma}_{11}^{(i)}\right)^{2}.  \end{array} $$

We substitute the estimates from Equations (15)-(19) back into Equations (9)-(14) to obtain the final hyperparameter estimates.

One must keep in mind that the calibration procedure explicitly assumes the MP model. Hence, one can only expect an improvement in the classification performance if the data follow the MP model.

### Computation

To obtain the MP OBC, we approximate the effective class conditional densities in order to minimize the expected error in a pointwise fashion:

(20) $$\begin{array}{@{}rcl@{}} p\left(\mathbf{x}|y,S_{n}\right)& =&\int_{\Theta_{y}}p\left(\mathbf{x}|y,\theta_{y}\right)p\left(\theta_{y}|S_{n}\right)d\theta_{y}  \\ & \approx& \frac{1}{M}\sum_{i=1}^{M}p\left(\mathbf{x}|y,\theta_{y}^{(i)}\right), \end{array} $$

where $\theta _{y}^{(i)}$ are *M* samples of *θ*_*y*_ from the model posterior distributions.

For clarity of presentation, we do not consider the class variable *y*, and we assume a single class. We do this because the computation can be performed per-class due to the assumed independence between the classes and the marginal probability, *p*(*c*,*θ*_0_,*θ*_1_)=*p*(*c*)*p*(*θ*_0_)*p*(*θ*_1_).

To obtain posterior samples of *θ* using the Metropolis Hastings MCMC algorithm we define a proposal distribution *p*(*θ*^′^|*θ*) to obtain a new value for the class parameters *θ*^′^ from the old values *θ*. We then calculate the acceptance ratio

$${} R\,=\,\min\! \left\{ \!1,\frac{p\left(\theta^{\prime }|S_{n}\right)p\left(\theta^{\prime }|\theta\right) }{p\left(\theta |S_{n}\right)p\left(\theta |\theta^{\prime}\right)}\!\right\} \,=\,\min\! \left\{ \!1, \frac{p\left(S_{n}|\theta^{\prime}\right)p\left(\theta^{\prime}\right)}{p\left(S_{n}|\theta\right)p\left(\theta \right)}\!\right\}, $$

under the assumption of a symmetric proposal distribution (*p*(*θ*^′^|*θ*)=*p*(*θ*|*θ*^′^)). The process of proposing and accepting samples from this distribution with the probability *R* induces a Markov chain. Positivity of the proposal distribution (*p*(*θ*^′^|*θ*)>0 for any *θ*) is a sufficient condition for ergodicity of this Markov chain. Furthermore, this Markov chain admits a steady-state distribution equal to our desired posterior distribution *p*(*θ*|*S*_*n*_) [34].

From the definition of the likelihood,

$$\begin{aligned} p\left(S_{n}|\theta\right)& =\prod_{i=1}^{n}p\left(\mathbf{x}_{i}|\theta\right)=\prod_{i=1}^{n}p\left(\mathbf{x}_{i}|\lambda_{i}\right) \\ & =\prod_{i=1}^{n}\prod_{d=1}^{D}p\left(x_{i,d}|\lambda_{i,d}\right), \end{aligned} $$

where *p*(**x**_*i*_|*θ*)=*p*(**x**_*i*_|*λ*_*i*_) owing to conditional independence. From the definition of the prior,

$$\begin{aligned} p(\theta) &= p(\mu, \Sigma, \lambda) \\ &= p(\lambda|\mu, \Sigma) p(\mu|\Sigma) p(\Sigma) \\ &= \prod_{i=1}^{n} p(\lambda_{i}|\mu, \Sigma) p(\mu|\Sigma) p(\Sigma). \end{aligned} $$

The posterior predictive distribution in (20) is approximated by

$$\begin{aligned} p\left(\mathbf{x}|S_{n}\right)& \approx \frac{1}{M}\sum_{i=1}^{M}p\left(\mathbf{x}|\theta^{(i)}\right) \\ & =\frac{1}{M}\sum_{i=1}^{M}\int_{\Lambda }p\left(\lambda |\theta^{(i)}\right) p\left(\mathbf{x}|\lambda \right)d\lambda \\ & =\frac{1}{M}\sum_{i=1}^{M}\int_{\Lambda }p\left(\lambda |\theta^{(i)}\right) \prod_{k=1}^{D}p\left(x_{k}|\lambda_{k}\right)d\lambda \\ & \approx \frac{1}{MT}\sum_{i=1}^{M}\sum_{g=1}^{T}\prod_{k=1}^{D}p\left(x_{k}| \lambda_{k}^{(g)}\right), \end{aligned} $$

where, *p*(**x**_*k*_|*λ*_*k*_)∼Poisson(*d*_*k*_ exp(*λ*_*k*_)), *λ*∼ Normal (*μ*,*Σ*), $\Lambda =\mathbb {R} ^{n\times D}$, and the *λ*^(*g*)^ are *T* vector-valued samples drawn from the appropriate class’s posterior distribution used to approximate the inner intractable integral. In addition, we use this approximation of the effective class-conditional density to calculate the conditional error estimates of (7) in a pointwise fashion.

Finally, because we have assumed a conjugate prior distribution for the marginal class probability *c*, the posterior expectation takes the closed form

$$\text{E}_{\theta |S_{n}}\left[c\right]=\frac{n_{0}+\alpha_{0}}{n_{0}+n_{1}+\alpha_{0}+\alpha_{1}}, $$

where the *n*_*y*_ are the number of training samples obtained from class *y* and the *α*_*y*_ are hyperparameters set to 1 for an uninformative prior. Conjugacy was used for this one parameter because the increased flexibility of the full sampling approach was deemed not necessary due to the constrained, univariate nature of the parameter. If more complex relationships between *c* and other parameters were desired, then a sampling approach using non-conjugate priors would be straightforward to implement.

### Synthetic data

To evaluate OBC performance in the setting of the MP model, we generate synthetic data using the method proposed in [35] to simulate gene expression/mRNA concentrations (see Additional file 1). These gene expression values are then statistically sampled to emulate modern sequencing machines as described in [21]. Parameter values are drawn from the following distributions to examine a wide variety of classification problems:

$$\begin{aligned} \mu_{y}& \sim \text{Normal}(0,0.2), \\ \sigma_{y}& \sim \text{Inverse-Gamma}(1,3), \\ \rho & =\text{Uniform}(0.0,1.0), \\ d_{\text{low}}& =9, \\ d_{\text{high}}& =11, \\ \text{blocksize}& =5. \end{aligned} $$

With these parameters, ten global, twenty heterogeneous, and ten non-marker features are generated. Then four features are randomly chosen to represent a mixture of features of various classification quality. Following [21], the features in the data are zero mean and unit standard deviation normalized except for the MP OBC. The exception occurs because the MP model expects features to be positive integers and normalization is not necessary. The discarded features are used for calibration of the MP OBC priors, and 3000 samples are generated from each class to estimate the true classification rate for each classifier.

We use four features in this synthetic data classification study owing to limited computational resources as discussed in Section ‘Computational limitations’.

The synthetic data generation method proposed in [35] imposes the strong assumption of a homogeneous covariance (HC) structure between the two classes of data. This assumption does not hold for biological situations where interactions between features are not necessarily preserved between classes, and this occurs frequently in biology when considering the possible effects of canalizing genes, nonlinear gene regulation, and mutations in the case of cancer [36,37]. Specifically, if the canalizing gene is not observed, and differs in activity between the two classes, then the measured correlation between two downstream genes could potentially be negligible in one class while strong in the other class. Similarly, for highly nonlinear gene regulation, if a gene in one class is in the saturation region of its response curve from a master gene, then the correlation will be low, while a lower expression level in the other class would allow for a large measured correlation with the same canalizing gene. And finally, if one class represents normal gene expression and the other tumor-related expression, then a correlation might exist from a functioning pathway in the normal tissue, but a mutation could result in a lack of correlation effects in the tumor.

Hence, we modify the synthetic data generation procedure in an attempt to produce synthetic datasets more representative of such nonlinear phenomena in biology. In this modified procedure, we allow independent covariance (IC) matrices for the features of the two classes. To generate these covariance matrices, *Σ*_*y*_, we utilize independent draws from inverse-Wishart distributions with parameters *κ*_*y*_=22,*D*=20, and scale matrix $S = {\sigma _{y}^{2}}$ (*κ*−1−*D*)*I*_*D*_. To examine the effects of feature correlation in IC datasets, we can also generate low-correlation covariance matrices by zeroing the off-diagonal terms. Once the covariance matrix for class *y* is obtained, location parameters for gene-expression values for each sample point are drawn from the respective multivariate normal distribution *λ*_*y*_∼*N*(*μ*_*y*_,*Σ*_*y*_). Each sample point is then assumed to be normalized through an upper quartile or other suitable method, but in practice any sample-based normalization is imperfect. We reflect this variation by drawing the sequencing depth *d*_*i*_ from a Uniform(*d*_low_,*d*_high_) distribution, giving the rate of the Poisson process as *d*_*i*_ exp*λ*_*i*_. The number of reads for a single gene from a single sample is then drawn from this Poisson distribution. See Additional file 1 for more detail.

The OBC is optimal on average across the space of distributions determined by its prior distributions. To avoid biasing the performance comparison, we draw the classification problem datasets using different distributions than those of the OBC priors. See Additional file 1 for more detail.

### Real data

We consider a real RNA-Seq dataset composed of level 3, RNASeqV2 data from the Cancer Genome Atlas (TCGA) project. It contains 484 and 470 specimens from lung adenocarcinoma and lung squamous cell carcinoma tumor biopsies, respectively. The samples are mapped read counts against 20531 known human RNA transcripts as generated by the University of North Carolina at Chapel Hill, one of the Genome Sequencing Centers for the TCGA. The data for each cancer type is the result of processing approximately 20 billion reads and the read count files for each are one gigabyte apiece. The problem is to classify the tumor types. Because the class-0 (lung adenocarcinoma) and class-1 (lung squamous cell carcinoma) sample sizes, 484 and 470, are not chosen randomly, we are confronted with the problem of separate sampling. This means that there is no way to obtain a posterior distribution for *c* and therefore *c* must be known in advance. Based upon records from 2006-2010, we have a very accurate estimate, 48,600/141,300≈0.34 [38]. Whereas we can use the value of *c* directly, along with all of the data, in designing the OBC, for classification rules that do not use *c* explicitly, the separately sampled data must be maximally subsampled to the proper sampling ratio *c* before applying the classification rule [39]. This means that for *N*_*trn*_ desired samples, the sample subsets will contain round((1−*c*)*N*_*trn*_) and round(*cN*_*trn*_) for class 0 and 1, respectively. Moreover, holdout error estimation, which we use here, must be properly adapted for separate sampling for all design methods, including the OBC. The holdout estimate is given by

$$\hat{\varepsilon}_{c}=c\hat{\varepsilon}_{0}+(1-c)\hat{\varepsilon}_{1}, $$

where $\hat {\varepsilon }_{0}$ and $\hat {\varepsilon }_{1}$ are the ordinary holdout estimators (performed on all remaining data samples not used for training) for the class-0 and class-1 errors, respectively [39]. We note that many studies have made the mistake of using classification rules designed for random sampling when sampling is separate. This can have devastating effects on classifier performance [39].

While averaging over sample subsets for holdout error estimation, we also average over uniformly, randomly selected gene subsets of size 4. This sampling occurs from low (1-10 average reads per gene) expression genes. We sample from these lower expression genes because we are ultimately interested in classification problems where the delineation between phenotypes is determined by genes with low expression. We used 10,000 for averaging in order to obtain a large enough sample over this feature and sample subset space to achieve repeatable results (data not shown). Computational runtime for each sample and gene subset was similar to the synthetic data.

## Results and discussion

The Additional file 1 contains a simple two-class, two-feature demonstration of the overall procedure to allow for easy visualization and interpretation. Here we discuss the results for the synthetic and real data.

### Synthetic data

**Figure 3:**
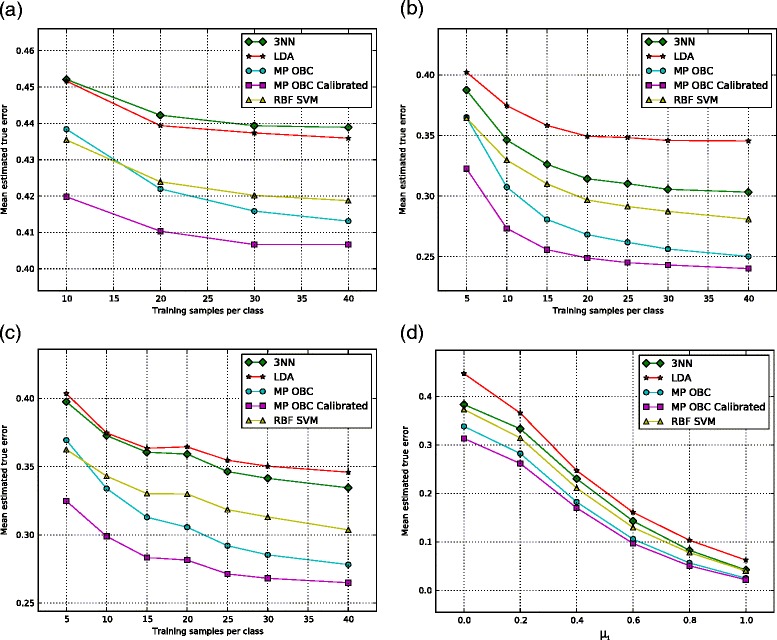
**Synthetic RNA-Seq classification.** Synthetic data classification results with **(a)** homogeneous-covariance, **(b)** high correlation independent-covariance, **(c)** low correlation independent-covariance, and **(d)** high correlation independent-covariance data at several problem difficulties.

To evaluate classification performance, classifiers were trained using 3NN, LDA, and c-support vector machines with a radial basis function kernel [22]. Starting with the homogeneous-covariance model, Figure 3a shows that the performance of the multivariate Poisson OBC is better than nonlinear SVM when more than 10 samples are available and is significantly better than any other classifier when using calibrated features. Equivalently, by using discarded features, we can obtain the same classification performance while requiring fewer training samples.

In the case of independent-covariance data with highly correlated features, Figure 3b shows superior classification performance of the MP OBC at nearly all sample sizes considered. In addition, for calibrated prior distributions, the performance of the MP OBC improves. This improvement is greater when the sample sizes are small, which demonstrates the importance of additional knowledge (through discarded features) when data are expensive to obtain or not readily available.

The superior performance of the OBC relative to LDA, 3NN, and SVM in Figure 3b is on account of classification optimization relative to the model, which characterizes prior information. To further investigate OBC improvement, we again considered heterogeneous covariance matrices but with independent features to determine if there is any difference in the relative performance between the classifiers. In fact, the results provided in Figure 3c show identical relative performance to the error curves in Figure 3b, thereby indicating that both the standard classifiers and the OBC, relative performance (at least in the case considered) is not affected by whether or not the features are correlated. Indeed, comparing Figure 3a with Figures 3b and 3c, we see that the relative performance of SVM, MP OBC, and calibrated MP OBC is the same in both the homogeneous and heterogeneous models. The switch in relative performance between LDA and 3NN between Figure 3a and Figures 3b and 3c is not surprising because LDA is optimal for a fixed (known) homogeneous Gaussian model but not for a heterogeneous Gaussian model.

The larger overall classification errors in Figure 3a as compared to Figures 3b and 3c are due to the different covariance matrices generated by the HC and IC models. Each model required different generating distributions for {*σ*_*y*_,*ρ*} and {*S*,*κ*} for the HC and IC cases, respectively, and the particular choices made in Section ‘Synthetic data’ resulted in larger dispersions and higher errors in the HC models than the IC models. To demonstrate this, we tested LDA with 1000 training and testing samples across 1000 random generating distributions and found the average HC classification error to be 0.41 and the IC error to be 0.32. This is despite LDA being optimal for homogeneous, fixed, known Gaussian cases and sub-optimal for heterogeneous, fixed, known Gaussian cases, where the former is similar to the HC case.

Still using independent-covariance data, we fixed the mean of class 0 at *μ*_0_=0.0 in Figure 3d, and varied *μ*_1_ from 0.0 to 1.0 to make the classification problem harder and easier, respectively. Across this range of classification problems, the MP OBC had better classification performance than the other classification methods. In addition, calibrated priors improved performance further, especially for harder classification problems.

### Real data

In Table 1, we chose ten genes at random from adenocarcinoma tumor TCGA samples and performed model diagnostics [31], p. 143, by calculating posterior predictive p-values for interquartile distance (IQR) as a measure of dispersion. In the Additional file 1, we provide additional test statistics and graphical predictive posterior model diagnostics. These results indicate that RNA-Seq overdispersion is modeled sufficiently with the MP model.

**Table 1:** **Posterior predictive model diagnostics are given for 10 randomly selected genes from adenocarcinoma TCGA samples**

**Gene ID**	**IQR (** ***S***_***n***_ **)**	**95% int. for IQR (** ***x*** ^***rep***^ **)**	**p-value**
UPK1A|11045	2.12	[1.0, 3.0]	0.09
OR4P4|81300	0.00	[0.0, 0.0]	0.50
PCDHA12|56137	139.22	[107.8, 187.0]	0.54
MDS2|259283	1.85	[2.0, 5.0]	1.00
AXIN2|8313	347.69	[331.5, 439.3]	0.85
DYNLT1|6993	848.41	[830.0, 1043.3]	0.90
RARA|5914	786.43	[706.8, 881.3]	0.62
TMEM194A|23306	396.06	[367.0, 471.3]	0.76
AGPS|8540	496.45	[505.8, 636.5]	0.97
NLRP2|55655	854.47	[381.3, 677.5]	0.00

Inter-quartile distance (IQR) is used as a robust measure of dispersion. In the table, *IQR(S*_*n*_ *)* is the training data’s IQR, followed by the 95-th credible interval, and the posterior predictive P-value. In cases where the P-value is close to 0 or 1, the true test statistic’s distance from the 95-th credible interval can be used to determine the magnitude of the mis-fit.

**Figure 4:**
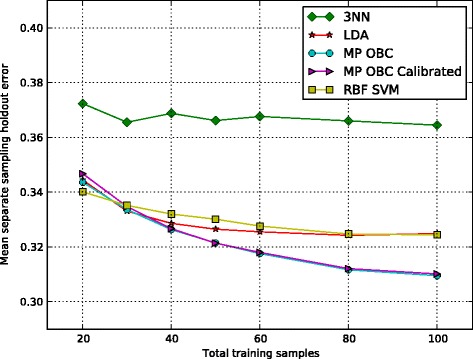
**TCGA RNA-Seq classification.** Average holdout errors were computed over 10,000 training sets and feature subsets using two types of lung cancer RNA-Seq data from TCGA. MP OBC with and without calibrated priors demonstrates superior performs across a range of training sample sizes. In addition, providing the MP OBC with calibrated priors does not appear to improve performance in this particular dataset.

In Figure 4, we see mean holdout errors averaged over 10,000 training sets and testing sets of TCGA data as described in Section ‘Real data’. Here the MP OBC performs better than all other classifiers across most training sample sizes considered, but calibration does not improve performance for this particular dataset. Recall that improvement owing to calibration depends on the extent to which the data satisfy the MP model. If the aim of this paper were to build an operational classifier based on the TCGA data, then we would have to go back and extensively study the data set to examine deviations from the model – for instance, outliers; however, here our aim is to show the functionality of the OBC with non-Gaussian data based on MCMC and apply it to the MP model. The fact that the MP OBC performs well on the real data satisfies this aim. Calibration is a tricky business and it would be a major separate study to characterize the manner in which model variation affects calibration, even if we were to perform an intensive study of this particular data set. Performance on the synthetic data demonstrates the effectiveness of the calibration when the model is satisfied.

### Computational limitations

The results in Figure 3 and Figure 4 required tens of thousands of MCMC runs. Owing to limited available computational resources, we could only allocate around 30 seconds on a single CPU core for each MCMC run. This necessitated using only four genes for these classification results as each iteration of the MCMC procedure has time complexity of *O*(*D*^3^), where *D* is the number of features. In practice, one would have a small number of data sets and could use parallel computing to devote more time and computing effort for the classification. For example, in timescales on the order of hours on a typical workstation, we have successfully performed classification using 50 genes.

The other classification methods compared in this study have smaller computational requirements and can correspondingly handle larger numbers of features given the same available resources. However, for the small sample sizes often available in biology, 50 genes is typically beyond the “peaking” point where most classifiers decrease in classification performance as more features are added (for a fixed number of training samples) [40]. Incidentally, the OBC does not suffer this “peaking phenomenon” as shown in [10].

In addition, the computational time requirements of classification is typically not a bottleneck in translational medicine given the timescales used in collecting biological data. In these settings, the accuracy of classification is much more valuable than rapid runtimes, and this is the primary advantage of the computational OBC framework proposed in this paper.

## Conclusions

We have demonstrated that Bayesian classification can be applied to specific problem domains such as RNA-Seq through statistical modeling and MCMC computation. The resulting classifier provides superior classification performance compared to state-of-the-art classifiers such as SVM with a radial basis kernel. Although we have not discussed error estimation – our interest in the present paper being classification, *ipso facto*, the MCMC approach to optimal Bayesian classification can be applied, via [6,7] and [12,13], to obtain optimal MMSE error estimators for any classification rule and sample-conditioned evaluation of the MSE for error estimation.

Future work includes examining the normalization parameter *d* and determining if additional performance improvements can be made by considering the distribution over *d* rather than transforming the original data through the process of data normalization. Additionally, more efficient computational techniques could be used to allow for larger feature sizes, including program optimization and utilizing structure in the feature covariance to reduce the size of the parameter space.

## Additional file

Additional file 1 **Supplementary Materials.** Algorithms and Model Diagnostics. Supporting details including in-depth algorithms and model diagnostic plots and figures are given in a single multi-page PDF.
