# Supplementary material for: MCMC implementation of the optimal Bayesian classifier for non-Gaussian models: model-based RNA-Seq classification
Source: BMC Bioinformatics. 2014 Dec 10;15(1):401. doi: 10.1186/s12859-014-0401-3 (PMC4265360; doi:10.1186/s12859-014-0401-3)
Supplement: Supplementary file 1 — Supplementary Materials. Algorithms and Model Diagnostics. Supporting details including in-depth algorithms and model diagnostic plots and figures are given in a single multi-page PDF. [file 12859_2014_401_MOESM1_ESM.pdf]

Additional File 1 - A Bayesian Multivariate Poisson Model for RNA-Seq Classification

December 3, 2014

Additional File 1: Algorithm 1 Calibrate Priors - This is the procedure to use discarded features to create calibrated prior distributions for use in a classification problem.

μ-list $\leftarrow$ []
$\Sigma_{i,i}$-list $\leftarrow$ []
$\Sigma_{i,j}$-list $\leftarrow$ []
for $i = 1 : s$ **do** $\triangleright$ s pairs sampled
    Randomly select a pair of features f_1, f_2
    $dsub \leftarrow$ data for this pair
    Initialize uniform priors
    MCMCSamples $\leftarrow$ N MCMC Samples using $dsub$
    $\mathrm{E}[\mu_1], \mathrm{E}[\mu_2] \leftarrow$ Sample μ means from MCMCSamples
    Append $\mathrm{E}[\mu_1], \mathrm{E}[\mu_2]$ to μ-list
    Append $\mathrm{E}[\Sigma_{1,1}], \mathrm{E}[\Sigma_{2,2}]$ to $\Sigma_{i,i}$-list
    Append $\mathrm{E}[\Sigma_{0,1}]$ to $\Sigma_{i,j}$-list
sigdiagmean $\leftarrow$ mean($\Sigma_{i,i}$-list)
sigoffmean $\leftarrow$ mean($\Sigma_{i,j}$-list)
sigdiagvar $\leftarrow \frac{1}{s-1}\sum_{i=1}^{s}(\text{sigdiagmean}-\Sigma_{i,i}\text{-list}[i])^2$
$\widehat{m} \leftarrow$ mean(μ-list)
$\widehat{\nu} \leftarrow$ var(μ-list)
$\widehat{\sigma^2} \leftarrow 2\times$ sigdiagmean $\times(\frac{\text{sigdiagmean}^2}{\text{sigdiagvar}} + 1)$
$\widehat{\rho} \leftarrow \frac{\text{sigoffmean}}{\text{sigdiagmean}}$
$\widehat{\kappa} \leftarrow \frac{2\times\text{sigdiagmean}^2}{\text{siagdiagvar}} + D + 3$

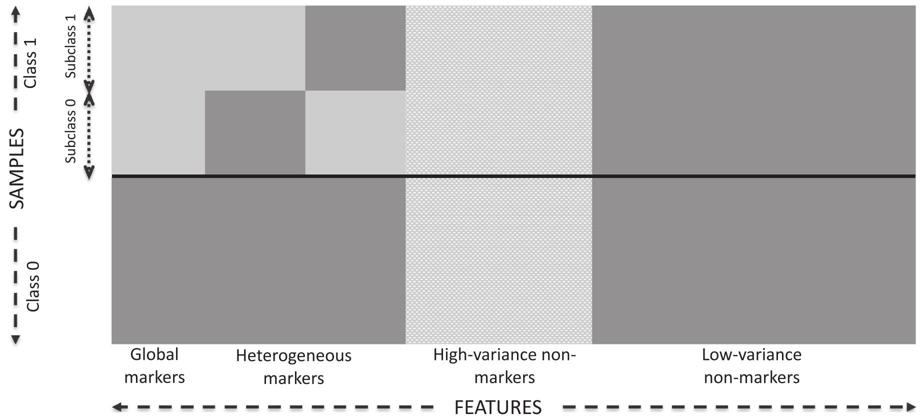

Additional file 1: Figure 1: The multivariate normal distribution used to generate samples for the IC synthetic data case. The block structure indicates the several different types of features that are generated. Used with permission from Ghaffari *et al.*, 2013.

Additional File 1: Algorithm 2 Generate IC Synthetic Data - To examine the effects of independent covariance matrices, we used the following IC method to first draw random covariance matrices for each class, and then to sample data.

Require: $N, \mathrm{d}_{low}, \mathrm{d}_{high}$ ▷ N: Number of samples desired

$D \leftarrow 20$
$\kappa \leftarrow D + 2$
for each class **do**
  $\mu \leftarrow \text{Normal}(0, 0.2) \times \text{ones}(D)$
  $\sigma \leftarrow \text{Normal}(0, 0.2)$
  $\Sigma \leftarrow \text{Inverse-Wishart}(I_D(\kappa - D - 1) * \sigma, D + 2)$
  if Low correlation features **then**
    off-diagonal$(\Sigma) \leftarrow 0$
  data $\leftarrow$ empty $N \times D$ matrix
  lams $\leftarrow$ Draw N vectors from Normal(μ, Σ)
  for $i = 1 : N$ **do**
    for $j = 1 : D$ **do**
      data$[i, j] \leftarrow$ Poisson-draw(Uniform$(\mathrm{d}_{low}, \mathrm{d}_{high}) \times \exp(\text{lams}[i, j])$)

Additional File 1: Algorithm 3 Synthetic Validation Procedure - The steps used to generate the sets of points for each N_{trn} (the number of training samples in each class along the x-axis in the paper's Figure 3.

for $i = 1 : N$ **do** ▷ N: Number of averages desired
  $\mu_0 \leftarrow$ Normal-draw$(0.0, 0.2)$
  $\mu_1 \leftarrow$ Normal-draw$(0.0, 0.2)$
  $\sigma_0 \leftarrow$ InverseGamma-draw$(3.0, 1.0)$
  $\sigma_1 \leftarrow$ InverseGamma-draw$(3.0, 1.0)$
  train-data-0 $\leftarrow$ genData$(\mu_0, \sigma_1, N_{trn}, \rho)$
  train-data-1 $\leftarrow$ genData$(\mu_1, \sigma_1, N_{trn}, \rho)$
  test-data-0 $\leftarrow$ genData$(\mu_0, \sigma_1, N_{test}, \rho)$
  test-data-1 $\leftarrow$ genData$(\mu_0, \sigma_1, N_{test}, \rho)$
  used-features $\leftarrow$ Randomly select 4 features
 Using Training data:
  hyperparameters $\leftarrow$ MCMC using Algorithm 1 and used-features[c]
  Train (Run) Calibrated MCMC with hyperparameters
  Train (Run) MCMC with weakly informative priors
  Train SVM
  Train LDA
  Train 3NN
  Train Normal OBC
 Using testing data:
  Evaluate Calibrated MCMC
  Evaluate MCMC with weakly informative priors
  Evaluate SVM
  Evaluate LDA
  Evaluate 3NN
  Evaluate Normal OBC

Additional File 1: Algorithm 4 Real Data Validation Procedure - The procedure used to generate each set of points along the x-axis of Figure 4 in the paper given a desired number of training samples $N_{trntotal}$ over N averages with an *a priori* known value of c.

for $i = 1 : N$ **do** ▷ N: Number of averages desired
    train-data$_0$ ← draw round($c * N_{trntotal}$) samples from data$_0$
    train-data$_1$ ← draw round($(1 - c) * N_{trntotal}$) samples from data$_1$
    test-data$_0$ ← data$_0$ - train-data$_0$
    test-data$_1$ ← data$_1$ - train-data$_1$
    used-features ← Randomly select 4 features
  Using Training data:
    hyperparameters ← Algorithm 1 MCMC using used-features^c
    Train (Run) Calibrated MCMC using hyperparameters
    Train (Run) MCMC with weakly informative priors
    Train SVM
    Train LDA
    Train 3NN
    Train Normal OBC
  Using testing data:
    Evaluate Calibrated MCMC
    Evaluate MCMC with weakly informative priors
    Evaluate SVM
    Evaluate LDA
    Evaluate 3NN
    Evaluate Normal OBC

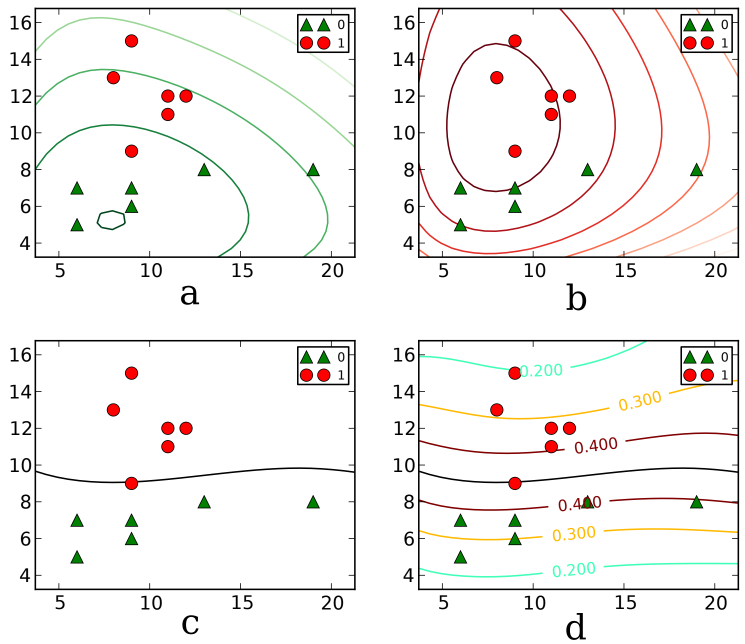

Additional file 1: Figure 2: A simple two class, two gene, synthetic example demonstrates the use of the MP OBC. Six training samples from each class (circles and triangles) are shown in all four panels and used to train the MP model. After MCMC computation, the resulting effective class conditional density contour is shown for the triangles in panel a and the circles in panel b. Panel c then shows the resulting MP OBC decision boundary resulting from these effective class conditional densities and panel d shows the contours of the optimal Bayes conditional error estimate plotted next to the classifier decision boundary.

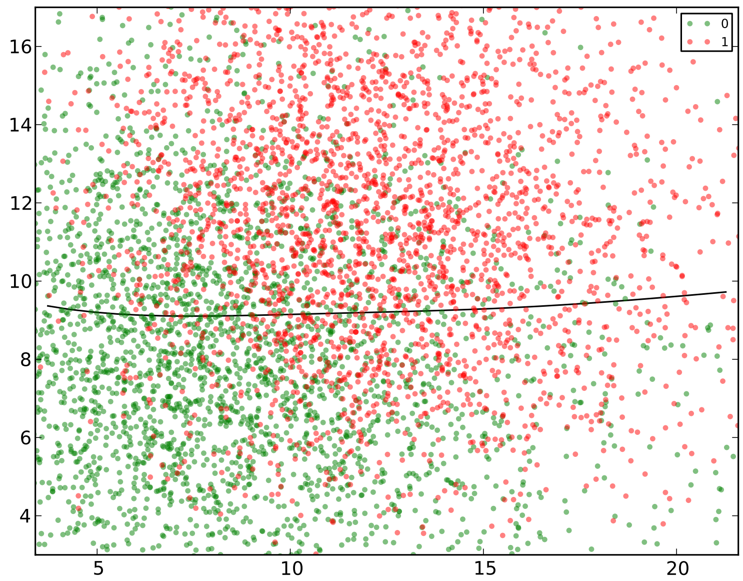

Additional file 1: Figure 3: Using the same classifier, we can now evaluate the performance of the classifier using 3000 testing samples from each class. When evaluated and averaged, this particular example results in a classification error of 0.29.

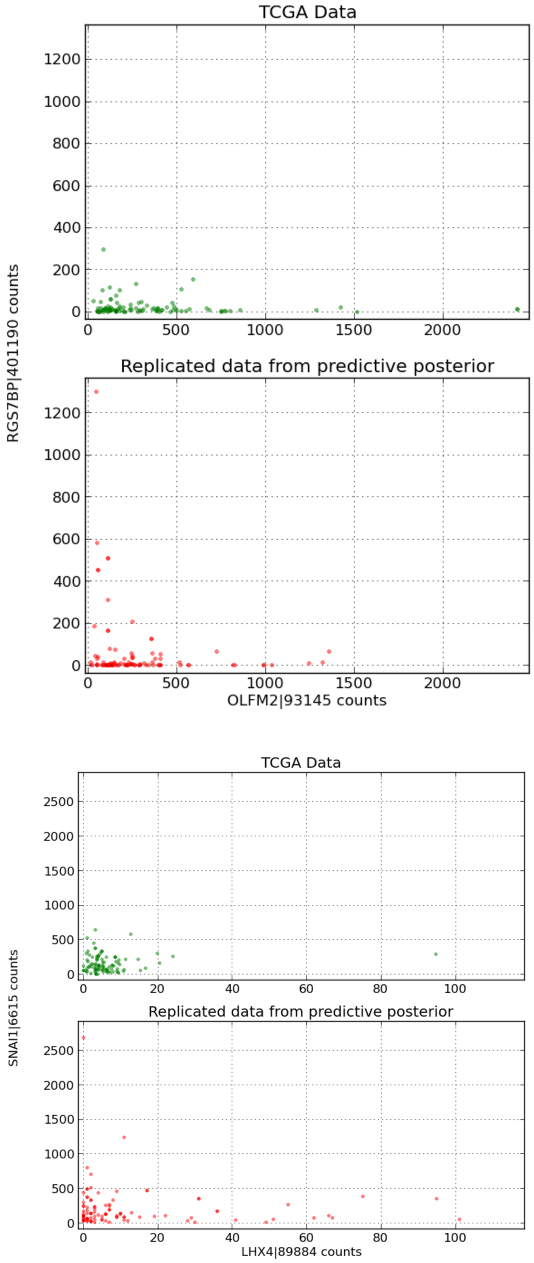

Additional file 1: Figure 4: Two examples of 100 samples from adenocarcinoma TCGA tumor samples and the posterior predictive x^{rep} simulation from the MP model.

Additional file 1: Table 1: Posterior predictive model diagnostic – 5th quantile.

Gene ID	Mean expression (counts)	$T(S_n)$	95th int. $T(x^{rep})$	P-value
MRGPRX1—259249	0.0	0.00	[0.00, 0.00]	0.50
C17orf105—284067	0.0	0.00	[0.00, 0.00]	0.50
HBBP1—3044	0.1	0.00	[0.00, 0.00]	0.50
SNORA18—677805	0.2	0.00	[0.00, 0.00]	0.50
SCN10A—6336	0.3	0.00	[0.00, 0.00]	0.50
CDCP2—200008	0.7	0.00	[0.00, 0.00]	0.50
FGF17—8822	3.3	0.00	[0.00, 0.00]	0.50
NTN5—126147	5.7	1.32	[0.00, 0.00]	0.00
NCRNA00185—55410	11.5	0.00	[0.00, 0.00]	0.50
CCR8—1237	16.6	1.46	[0.00, 1.90]	0.06
CCDC33—80125	23.1	0.00	[0.00, 0.00]	0.50
PPAPDC3—84814	23.5	6.00	[2.95, 8.95]	0.32
PCDHGB2—56103	68.1	5.19	[1.95, 10.80]	0.47
FAM81A—145773	81.1	13.38	[5.00, 17.90]	0.23
ZNF383—163087	81.2	54.44	[24.00, 48.45]	0.01
ANKRD1—27063	87.1	1.23	[0.00, 3.95]	0.52
UGT2B4—7363	93.4	0.00	[0.00, 0.00]	0.50
IL12RB1—3594	98.9	15.33	[13.85, 36.40]	0.88
ZNF628—89887	160.0	75.54	[41.95, 83.80]	0.16
FBF1—85302	184.2	52.03	[29.60, 75.50]	0.41
ZNF615—284370	209.2	70.60	[36.95, 81.40]	0.16
RHBDD1—84236	299.9	127.69	[82.90, 170.90]	0.46
NICN1—84276	330.1	173.29	[87.95, 187.40]	0.12
COQ6—51004	369.7	193.25	[106.95, 194.20]	0.05
CHAF1A—10036	387.3	150.70	[81.55, 190.65]	0.30
DTD1—92675	534.5	273.70	[141.50, 282.20]	0.07
EARS2—124454	663.7	380.07	[193.10, 356.85]	0.03
KIAA1737—85457	668.3	365.58	[214.25, 393.95]	0.12
LRRC8D—55144	690.2	445.36	[214.45, 405.95]	0.02
SKIL—6498	691.0	336.66	[199.70, 368.85]	0.15
WDR36—134430	761.6	531.67	[258.20, 479.55]	0.02
ZNF259—8882	831.0	455.39	[221.15, 425.20]	0.03
CHSY1—22856	1029.7	474.64	[269.05, 548.10]	0.15
DHX8—1659	1192.9	695.25	[354.10, 728.70]	0.08
AGTRAP—57085	1254.0	539.55	[283.75, 622.85]	0.20
VPS26B—112936	1337.3	643.79	[350.35, 716.25]	0.17
MCM4—4173	1543.3	343.29	[202.80, 528.50]	0.51
SLC2A3—6515	1559.7	338.07	[178.00, 474.20]	0.38
VPS39—23339	1594.0	908.35	[460.90, 964.45]	0.07
FOXA1—3169	1800.3	396.41	[217.10, 584.20]	0.40

Additional file 1: Table 2: Posterior predictive model diagnostic – Median.

Gene ID	Mean expression (counts)	$T(S_n)$	95th int. $T(x^{rep})$	P-value
MRGPRX1—259249	0.0	0.00	[0.00, 0.00]	0.50
C17orf105—284067	0.0	0.00	[0.00, 0.00]	0.50
HBBP1—3044	0.1	0.00	[0.00, 0.00]	0.50
SNORA18—677805	0.2	0.00	[0.00, 0.00]	0.50
SCN10A—6336	0.3	0.00	[0.00, 0.00]	0.50
CDCP2—200008	0.7	0.00	[0.00, 0.00]	0.50
FGF17—8822	3.3	1.01	[0.00, 1.00]	0.03
NTN5—126147	5.7	4.35	[2.00, 7.50]	0.41
NCRNA00185—55410	11.5	0.00	[0.00, 0.00]	0.50
CCR8—1237	16.6	12.18	[4.00, 17.50]	0.22
CCDC33—80125	23.1	6.01	[1.00, 10.00]	0.17
PPAPDC3—84814	23.5	19.68	[13.50, 24.00]	0.36
PCDHGB2—56103	68.1	33.59	[18.50, 46.00]	0.34
FAM81A—145773	81.1	45.82	[31.00, 71.50]	0.59
ZNF383—163087	81.2	85.50	[68.00, 104.50]	0.45
ANKRD1—27063	87.1	20.69	[9.50, 34.00]	0.36
UGT2B4—7363	93.4	1.77	[0.00, 4.00]	0.30
IL12RB1—3594	98.9	80.38	[56.50, 110.00]	0.45
ZNF628—89887	160.0	158.95	[121.00, 194.50]	0.39
FBF1—85302	184.2	162.49	[111.00, 211.00]	0.28
ZNF615—284370	209.2	184.09	[126.00, 218.00]	0.25
RHBDD1—84236	299.9	275.19	[214.00, 337.50]	0.48
NICN1—84276	330.1	320.44	[264.00, 436.00]	0.60
COQ6—51004	369.7	335.85	[265.00, 405.00]	0.46
CHAF1A—10036	387.3	302.09	[252.50, 452.50]	0.75
DTD1—92675	534.5	523.20	[385.00, 626.00]	0.30
EARS2—124454	663.7	603.62	[485.00, 732.50]	0.41
KIAA1737—85457	668.3	676.88	[529.00, 806.50]	0.39
LRRC8D—55144	690.2	645.04	[550.50, 835.00]	0.67
SKIL—6498	691.0	594.36	[479.50, 761.00]	0.56
WDR36—134430	761.6	752.27	[631.50, 909.00]	0.55
ZNF259—8882	831.0	658.89	[551.00, 903.00]	0.71
CHSY1—22856	1029.7	872.60	[721.00, 1146.50]	0.59
DHX8—1659	1192.9	1150.34	[957.00, 1552.00]	0.67
AGTRAP—57085	1254.0	1069.75	[839.00, 1375.50]	0.55
VPS26B—112936	1337.3	1189.98	[912.00, 1415.00]	0.40
MCM4—4173	1543.3	1094.04	[795.00, 1417.00]	0.41
SLC2A3—6515	1559.7	1053.06	[719.50, 1420.50]	0.45
VPS39—23339	1594.0	1651.32	[1274.00, 2078.50]	0.43
FOXA1—3169	1800.3	1168.83	[887.50, 1642.00]	0.62

Additional file 1: Table 3: Posterior predictive model diagnostic – 95th quantile.

Gene ID	Mean expression (counts)	$T(S_n)$	95th int. $T(x^{rep})$	P-value
MRGPRX1—259249	0.0	0.00	[0.00, 0.00]	0.50
C17orf105—284067	0.0	1.01	[0.00, 0.05]	0.00
HBBP1—3044	0.1	0.50	[0.00, 1.00]	0.07
SNORA18—677805	0.2	0.25	[0.00, 0.00]	0.00
SCN10A—6336	0.3	1.49	[0.05, 7.45]	0.38
CDCP2—200008	0.7	2.12	[0.05, 25.10]	0.56
FGF17—8822	3.3	16.60	[6.35, 279.40]	0.78
NTN5—126147	5.7	14.10	[16.40, 109.85]	0.97
NCRNA00185—55410	11.5	38.88	[1.05, 146.00]	0.20
CCR8—1237	16.6	38.64	[42.30, 308.40]	0.97
CCDC33—80125	23.1	107.94	[59.25, 2444.60]	0.85
PPAPDC3—84814	23.5	48.09	[35.05, 87.75]	0.66
PCDHGB2—56103	68.1	179.96	[89.40, 276.50]	0.36
FAM81A—145773	81.1	200.17	[127.60, 378.45]	0.54
ZNF383—163087	81.2	144.50	[144.55, 263.35]	0.95
ANKRD1—27063	87.1	225.79	[82.90, 400.90]	0.31
UGT2B4—7363	93.4	54.66	[19.50, 1361.35]	0.80
IL12RB1—3594	98.9	230.41	[155.80, 432.00]	0.65
ZNF628—89887	160.0	299.84	[269.00, 554.20]	0.86
FBF1—85302	184.2	374.78	[297.80, 748.75]	0.72
ZNF615—284370	209.2	368.14	[316.55, 769.65]	0.87
RHBDD1—84236	299.9	427.40	[455.05, 820.10]	0.97
NICN1—84276	330.1	737.03	[611.90, 1253.15]	0.74
COQ6—51004	369.7	568.48	[542.15, 1066.55]	0.90
CHAF1A—10036	387.3	834.67	[617.75, 1323.90]	0.54
DTD1—92675	534.5	989.05	[832.60, 1590.50]	0.75
EARS2—124454	663.7	1005.24	[970.90, 1902.00]	0.91
KIAA1737—85457	668.3	887.21	[1075.65, 1961.85]	1.00
LRRC8D—55144	690.2	1086.87	[1088.05, 2025.85]	0.95
SKIL—6498	691.0	1140.47	[1019.95, 2039.50]	0.80
WDR36—134430	761.6	1220.71	[1231.45, 2132.60]	0.95
ZNF259—8882	831.0	1169.19	[1186.25, 2164.80]	0.95
CHSY1—22856	1029.7	1705.17	[1557.95, 2971.50]	0.86
DHX8—1659	1192.9	1953.12	[2051.05, 4246.15]	0.97
AGTRAP—57085	1254.0	2240.63	[1841.20, 4096.95]	0.76
VPS26B—112936	1337.3	1847.39	[1949.45, 3436.10]	0.97
MCM4—4173	1543.3	3410.08	[2205.70, 5370.80]	0.41
SLC2A3—6515	1559.7	3378.11	[2183.05, 5712.85]	0.51
VPS39—23339	1594.0	2514.10	[2719.90, 5625.80]	0.98
FOXA1—3169	1800.3	3302.97	[2553.55, 6376.40]	0.73

Additional file 1: Table 4: Posterior predictive model diagnostic – IQR.

Gene ID	Mean expression (counts)	$T(S_n)$	95th int. $T(x^{rep})$	P-value
MRGPRX1—259249	0.0	0.00	[0.00, 0.00]	0.50
C17orf105—284067	0.0	0.00	[0.00, 0.00]	0.50
HBBP1—3044	0.1	0.00	[0.00, 0.00]	0.50
SNORA18—677805	0.2	0.00	[0.00, 0.00]	0.50
SCN10A—6336	0.3	0.50	[0.00, 0.00]	0.03
CDCP2—200008	0.7	0.98	[0.00, 1.00]	0.07
FGF17—8822	3.3	2.59	[1.00, 9.00]	0.52
NTN5—126147	5.7	6.93	[4.50, 21.75]	0.75
NCRNA00185—55410	11.5	5.45	[0.00, 1.50]	0.00
CCR8—1237	16.6	17.69	[11.00, 58.00]	0.66
CCDC33—80125	23.1	17.19	[6.25, 93.25]	0.60
PPAPDC3—84814	23.5	13.91	[11.00, 25.50]	0.79
PCDHGB2—56103	68.1	43.70	[28.50, 82.25]	0.60
FAM81A—145773	81.1	63.53	[38.75, 109.75]	0.51
ZNF383—163087	81.2	34.43	[40.25, 86.75]	1.00
ANKRD1—27063	87.1	74.52	[21.25, 83.75]	0.11
UGT2B4—7363	93.4	6.98	[2.00, 43.50]	0.64
IL12RB1—3594	98.9	84.17	[47.50, 130.00]	0.42
ZNF628—89887	160.0	100.43	[78.50, 171.50]	0.73
FBF1—85302	184.2	154.54	[95.00, 226.75]	0.36
ZNF615—284370	209.2	117.16	[96.00, 228.50]	0.82
RHBDD1—84236	299.9	126.20	[128.75, 275.50]	0.96
NICN1—84276	330.1	246.20	[179.75, 411.50]	0.61
COQ6—51004	369.7	153.72	[153.50, 361.00]	0.95
CHAF1A—10036	387.3	234.48	[172.75, 422.25]	0.72
DTD1—92675	534.5	297.30	[236.75, 533.75]	0.77
EARS2—124454	663.7	237.65	[262.25, 578.50]	0.98
KIAA1737—85457	668.3	220.32	[281.50, 651.75]	0.99
LRRC8D—55144	690.2	359.07	[285.00, 655.50]	0.83
SKIL—6498	691.0	247.07	[290.75, 615.75]	1.00
WDR36—134430	761.6	216.40	[331.25, 737.50]	1.00
ZNF259—8882	831.0	322.44	[314.50, 715.00]	0.94
CHSY1—22856	1029.7	441.38	[442.25, 1005.00]	0.95
DHX8—1659	1192.9	582.12	[584.75, 1240.00]	0.95
AGTRAP—57085	1254.0	622.58	[532.25, 1264.50]	0.86
VPS26B—112936	1337.3	529.40	[536.00, 1111.25]	0.96
MCM4—4173	1543.3	835.39	[631.25, 1588.50]	0.74
SLC2A3—6515	1559.7	845.02	[611.00, 1788.75]	0.79
VPS39—23339	1594.0	586.40	[764.75, 1831.00]	1.00
FOXA1—3169	1800.3	1275.06	[765.00, 2040.75]	0.50

Additional file 1: Table 5: Posterior predictive model diagnostic – Variance.

Gene ID	Mean expression (counts)	$T(S_n)$	95th int. $T(x^{rep})$	P-value
MRGPRX1—259249	0.0	0.00	[0.00, 0.00]	0.04
C17orf105—284067	0.0	0.11	[0.00, 0.12]	0.07
HBBP1—3044	0.1	0.08	[0.00, 0.25]	0.13
SNORA18—677805	0.2	0.01	[0.00, 0.05]	0.25
SCN10A—6336	0.3	0.36	[0.05, 1242.64]	0.69
CDCP2—200008	0.7	0.74	[0.16, 5117.85]	0.80
FGF17—8822	3.3	26.34	[32.90, 1109445.84]	0.97
NTN5—126147	5.7	42.93	[51.13, 8446.14]	0.96
NCRNA00185—55410	11.5	303.63	[1.35, 1340191.71]	0.53
CCR8—1237	16.6	157.20	[285.39, 81521.57]	0.98
CCDC33—80125	23.1	14201.11	[2292.16, 31223489.92]	0.79
PPAPDC3—84814	23.5	181.37	[112.92, 1048.31]	0.80
PCDHGB2—56103	68.1	4048.26	[1228.20, 20426.96]	0.52
FAM81A—145773	81.1	5661.96	[1970.14, 26450.20]	0.53
ZNF383—163087	81.2	1206.74	[1301.43, 6287.70]	0.96
ANKRD1—27063	87.1	9884.12	[1356.27, 51853.87]	0.36
UGT2B4—7363	93.4	5664.11	[291.72, 8368761.19]	0.66
IL12RB1—3594	98.9	4745.05	[2290.87, 27420.28]	0.79
ZNF628—89887	160.0	6534.92	[5024.43, 31689.22]	0.87
FBF1—85302	184.2	12711.43	[7286.06, 71120.08]	0.77
ZNF615—284370	209.2	11122.81	[9659.19, 69434.34]	0.88
RHBDD1—84236	299.9	8546.28	[13399.90, 63578.81]	0.99
NICN1—84276	330.1	32463.87	[26378.50, 154304.78]	0.87
COQ6—51004	369.7	15005.15	[19147.05, 94982.52]	0.98
CHAF1A—10036	387.3	47233.01	[28450.33, 196129.54]	0.74
DTD1—92675	534.5	59301.87	[45681.73, 251258.48]	0.90
EARS2—124454	663.7	52839.46	[58542.64, 301221.33]	0.98
KIAA1737—85457	668.3	48505.67	[75259.02, 340071.74]	0.99
LRRC8D—55144	690.2	48918.16	[78779.94, 378872.68]	1.00
SKIL—6498	691.0	56797.55	[69545.32, 366715.84]	0.97
WDR36—134430	761.6	49947.90	[88525.22, 422834.77]	1.00
ZNF259—8882	831.0	88575.80	[82583.27, 472063.68]	0.94
CHSY1—22856	1029.7	201986.35	[168564.65, 873474.11]	0.90
DHX8—1659	1192.9	348984.33	[285590.39, 1578955.67]	0.87
AGTRAP—57085	1254.0	424287.54	[248618.71, 1869240.10]	0.71
VPS26B—112936	1337.3	158070.60	[240558.63, 1169889.12]	1.00
MCM4—4173	1543.3	901681.93	[386142.75, 3614045.91]	0.63
SLC2A3—6515	1559.7	1037093.04	[421036.96, 4698669.95]	0.61
VPS39—23339	1594.0	328769.35	[481155.30, 2674633.59]	1.00
FOXA1—3169	1800.3	1688527.66	[680898.40, 7470155.18]	0.54
